# Resource selection by Sarda cattle in a Mediterranean silvopastoral system

**DOI:** 10.3389/fvets.2024.1348736

**Published:** 2024-03-07

**Authors:** Marco Acciaro, Marco Pittarello, Mauro Decandia, Maria Sitzia, Valeria Giovanetti, Giampiero Lombardi, Patrick E. Clark

**Affiliations:** ^1^AGRIS Sardegna, Sassari, Italy; ^2^Department of Veterinary Sciences, University of Torino, Grugliasco, Italy; ^3^Department of Agricultural, Forest and Food Sciences, University of Torino, Grugliasco, Italy; ^4^US Department of Agriculture-Agriculture Research Service, Northwest Watershed Research Center, Boise, ID, United States

**Keywords:** Mediterranean woodland types, cattle grazing, spatial behavior, global positioning system (GPS), space use

## Abstract

Knowledge of how grazing cattle utilize heterogeneous landscapes in Mediterranean silvopastoral areas is scarce. Global positioning systems (GPS) to track animals, together with geographic information systems (GIS), can relate animal distribution to landscape features. With the aim to develop a general spatial model that provides accurate prediction of cattle resource selection patterns within a Mediterranean mountainous silvopastoral area, free-roaming Sarda cows were fitted with GPS collars to track their spatial behaviors. Resource selection function models (RSF) were developed to estimate the probability of resource use as a function of environmental variables. A set of over 500 candidate RSF models, composed of up to five environmental predictor variables, were fitted to data. To identify a final model providing a robust prediction of cattle resource selection pattern across the different seasons, the 10 best models (ranked on the basis of the AIC score) were fitted to seasonal data. Prediction performance of the models was evaluated with a Spearman correlation analysis using the GPS position data sets previously reserved for model validation. The final model emphasized that watering point, elevation, and distance to fences were important factors affecting cattle resource-selection patterns. The prediction performances (as Spearman rank correlation scores) of the final model, when fitted to each season, ranged between 0.7 and 0.94. The cows were more likely to select areas lower in elevation and farther from the watering point in winter than in summer (693 ± 1 m and 847 ± 13 m vs. 707 ± 1 m and 635 ± 21 m, respectively), and in spring opted for the areas furthest from the water (963 ± 12). Although caution should be exercised in generalizing to other silvopastoral areas, the satisfactory Spearman correlations scores from the final RSF model applied to different seasons indicate resource selection function is a powerful predictive model. The relative importance of the individual predictors within the model varied among the different seasons, demonstrating the RSF model’s ability to interpret changes in animal behavior at different times of the year. The RSF model has proven to be a useful tool to interpret the spatial behaviors of cows grazing in Mediterranean silvopastoral areas and could therefore be helpful in managing and preserving ecosystem services of these areas.

## Introduction

Wooded grasslands are the most widespread agroforestry systems in Europe (1). By the term wooded grasslands, we mean a continuum where tree and/or shrub cover is dense enough to approach woodland or shrubland status on one end of the continuum, and sparse enough to approach grassland at the other end. Systems that combine grazing animals and trees are often called silvopastoral systems. Domestic ruminants have been grazing mountainous Mediterranean oak (*Quercus* spp.) woodlands for up to 4,500 years (2, 3). Various ecosystem services, including food (livestock) production, plant and animal diversity, carbon storage, nutrient cycling, regulation of climate, as well as esthetic and recreational values, are provided by Mediterranean silvopastoral activities on wooded grasslands (4–7). Moreover, Mediterranean Quercus-based silvopastoral systems are recognized as *a priori*ty by the Habitats Directive (Council Directive 92/43/EEC). Wooded grasslands derived from human-induced transformation of forests and, similar to other man-made ecosystems, the conservation of these silvopastoral systems and their ecological integrity is affected by the sustainability of animal production systems relying on pasture (8). Some authors (1, 9) point out that the anthropogenic origin of wood-pastures implies a need for constant and specific management and that livestock grazing is the most influential and dominant management intervention for the structure and dynamics of wood-pastures. Grazing activity and spatial distribution of the cattle are influenced by the patchy structure characterizing Mediterranean oak woodlands. Limiting the grazing pressure and selecting the grazing regime, among other practices, are important for ensuring tree regeneration while preventing the encroachment of dense shrub cover. To this end, a better understanding of how grazing animals utilize these heterogeneous landscapes and the factors involved is required (10–13). Free-roaming animals, such as cattle under continuous grazing systems in mountain pastures (14), especially if not rationally managed, could result in an uneven spatial distribution (15) with excessive grazing pressure in some areas, which then may suffer degradation, while leaving other areas underutilized. These areas often evolve toward shrub- and tree-encroached communities, with reduced accessibility, increased fire risk, loss of biodiversity (4, 16), and lower efficiency of forage utilization, with negative effects on ecosystem services (7) and erosion of the ecological and social–cultural values of wood-pastures. In the southern parts of Europe, overgrazing and wood overexploitation are recognized to be among the most important drivers of wood-pasture loss, with lack of tree regeneration, sometimes followed by a complete disappearance of vegetation and subsequent soil erosion (9, 17). In a recent report on the current status and future prospects of European terrestrial habitats,^1^ 5% of forests are classified as endangered, 24% as vulnerable, and 24% near threatened; the European Commission itself has identified overgrazing “as a major threat especially in several Mediterranean woodland types.” The capacity to understand and predict livestock spatial behavior would be a potent tool for the management and study of extensive grazing systems, as well as sustainable landscape management (16, 18–20). Nevertheless, knowledge of the behavior patterns of cows in Mediterranean silvopastural areas, and of their spatial and temporal distributions, is scarce because of the logistical constraints on monitoring animals that often cannot be easily observed. Therefore, an increased knowledge of the spatial behavior of grazing cows could play an important role to determine, e.g., if cattle exhibited more recursive use of certain areas during specific seasons than others, and if this behavior might then be mitigated by changing the grazing regime, watering points, or other managerial factors.

In order to study the landscape use by grazing animals, various records of the animals’ locations over time are required. Global positioning systems (GPS) to track animals together with geographic information systems (GIS) made it possible to relate animal distribution to landscape features (21, 22). The affordability of modern GPS tracking collars allows for accurate and consistent measurement of the distribution of livestock in the landscape, which helps evaluate different aspects of grazing management (23). Uniform grazing distribution is a major objective of grazing management practices (24, 25), and a knowledge of environmental characteristics affecting distribution is crucial to manage resource selection patterns of grazing animals (15, 26, 27). Several environmental factors affect livestock behavior and consequent resource selection patterns. These factors include distance to water (24, 28), composition of plant communities (29), degree of slope (30), and dense woody vegetation (31). Even the season is an important factor in the resource selection patterns of herbivores (32), being related to the length of daylight, which, according to the antipredator theory (33), impacts foraging behavior.

The beef livestock system in Sardinia (Italy) and other mediterranean regions is based on the suckler-cow system (34–36): the cows, mainly belonging to autochthonous breeds, are characterized by a good maternal aptitude and ability to exploit natural forage resources. Cow-calf pairs normally graze the mountainous and hilly areas, often with the presence of trees (17, 37–39). These silvopastoral systems (SPS) are commonly identified as a source of ecosystem services (Ess), including the provision of beef meat, wildlife habitat, and biodiversity and watershed protection (6, 40, 41). SPS services have found concrete application in the case of the recent certification of a Mediterranean silvopastoral area managed by AGRIS Sardegna and grazed by Sarda cattle (Monte Sant’Antonio, Macomer, NU), which is representative of the Mediterranean beef livestock system, according to FSC^®^ standards. The present paper reports a case study conducted the Monte Sant’Antonio, with the intention of enhancing the knowledge regarding spatial distribution and consequent resource selection patterns of mature Sarda cows and the most important determinants. Specific objectives of the present study are to: (i) develop a general spatial model or resource selection function (RSF) that provides accurate and robust predictions of cattle distribution patterns within an extensive Mediterranean mountainous silvopastoral area and (ii) determine whether the general RSF model provides an accurate prediction of cattle resource selection across seasons, interpreting changes in the spatial distribution of animals related to different seasons (*sensu lato*) and showing thus greater utility and impact (final model).

## Materials and methods

### Experimental site

**Figure 1:**
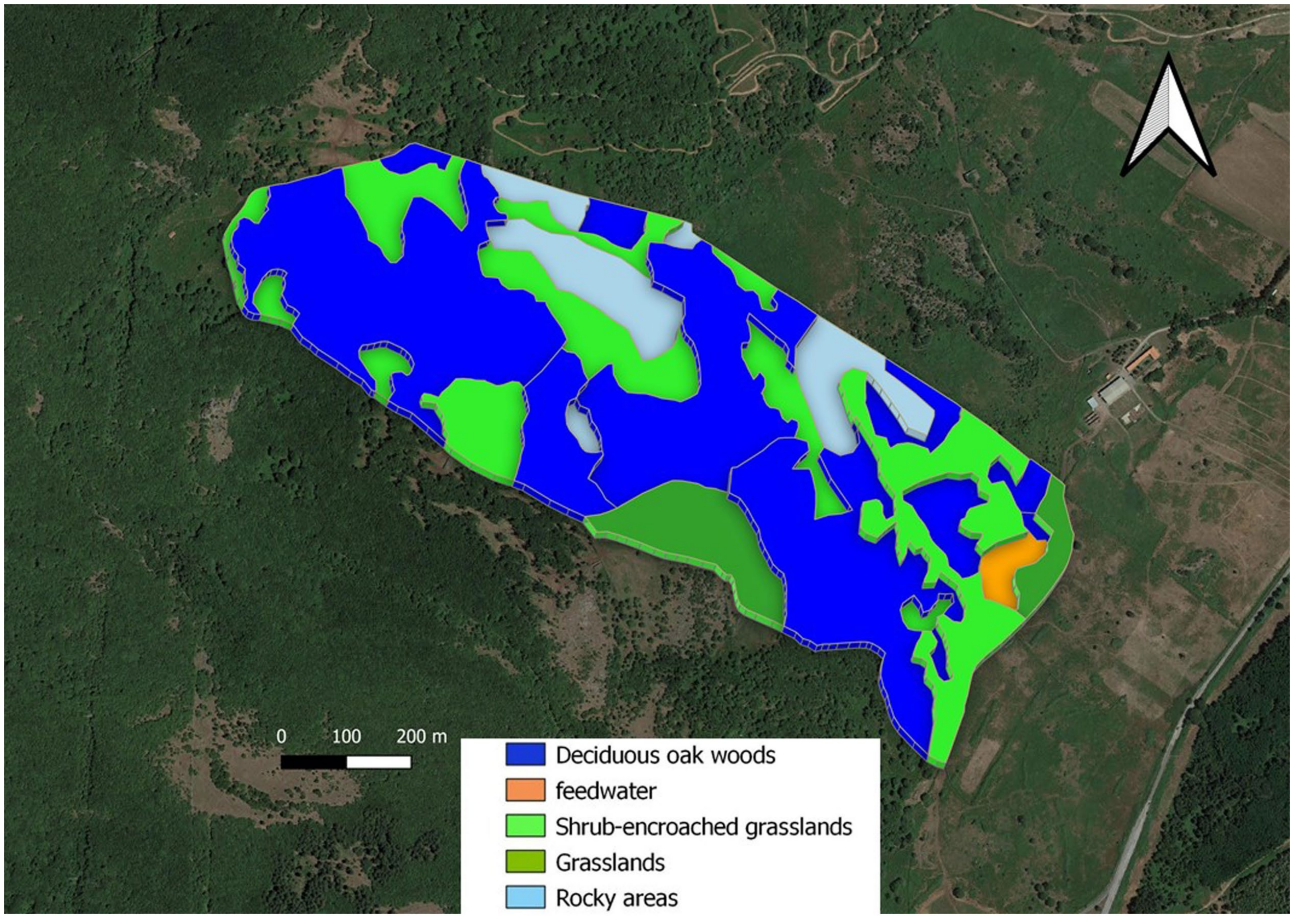
Map of experimental area. I. Deciduous oak woods, dark blue area; II. Shrub-encroached grasslands, bright green area; III. Grasslands, olive green area; IV. Rocky areas, grey; V. Feedwater area, orange from (42).

This study was conducted on the experimental farm of the Agricultural Research Agency of Sardinia (AGRIS Sardegna, Macomer, Italy), located in Monte Sant’Antonio (40°14′10′‘N, 8°42′31′‘E., Macomer, Italy). All experimental procedures were approved by the Ethical Committee on Animal Experimentation (OPBA, No. 2190/2019). The study area (Figures 1, 54 ha) is a fenced rangeland pasture with topography characterized by an average elevation of 695 m (range: 730–660 m above sea level) and an average slope of 10.7% (range: 0.5–36.3%). The climate is Mediterranean. Daily maximum, minimum, and mean air temperatures (2015–2022) at the weather station inside the experimental farm were 18.9, 7.9, and 13.2°C, respectively. Mean annual precipitation (2015–2022) was 576 mm (25/01/2022).^2^ Daily maximum, minimum, and mean air temperatures and precipitation events that occurred during the experimental periods are shown in Table 1. Between the first and second years of experimental data collection, a tornado struck in the western part of the experimental area. This event created grazeable clearings.

**Table 1:** Maximum (T max), minimum (T min), mean air temperatures (T avg.; mean ± s.d.) and rainfall patterns (total rainfall, mm) during the seven sampling periods of experiment (see below).

Season	Experimental periods	T max	T min	T avg.	Rainfall
Winter	26/02/2019 to 03/03/2019	13.6 ± 1.2	2.2 ± 1.6	7.8 ± 0.8	0.2
	14/12/2020 to 17/01/2021	9.0 ± 3.3	3.0 ± 2.7	5.9 ± 2.9	248.4
Spring	06/04/2019 to 18/04/2019	16.6 ± 2.5	4.3 ± 1.9	8.5 ± 2.0	52.4
	03/06/2020 to 08/07/2020	25.6 ± 4.5	12.3 ± 2.4	18.8 ± 3.4	35.6
Summer	16/07/2019 to 24/07/2019	30.3 ± 2.7	15.6 ± 3.2	22.8 ± 2.7	6.8
	05/08/2020 to 16/09/2020	29.0 ± 3.7	15.1 ± 2.6	21.7 ± 3.0	54.4
Autumn	26/09/2019 to 16/10/2019	22.0 ± 2.5	10.9 ± 1.9	15.9 ± 1.90	14.2

In the experimental area (Figure 1), we identified (see below):

- three vegetation units (*sensu stricto*), identified on a physiognomic-structural basis: (I) deciduous oak woods dominated by *Quercus pubescens* s.l.; (II) shrub-encroached grasslands dominated by *Rubus hulmifolius* and *Pteridium aquilinum* (bracken), with a sporadic presence of trees; and (III) grasslands dominated by grasses and other herbaceous species (e.g., forbs and legumes). No assessments of the homogeneity of forage production within units were carried out during the experiment.
- rocky areas, where rock covers more than 30% of the surface (IV);
- feedwater area around the water point and the feed supplement, the latter consisting of occasionally administered hay, mainly in winter (V).

The three vegetation units, the rocky area, and the feedwater area were determined by means of GIS thematic layers (see below). There is only one water point within the study area, located in the southern part of the experimental paddock. A strong preference for the feedwater area was expected, but we considered this area in the analyses to verify its role in cow spatial distribution.

The experimental area covered 54.3 ha, of which more than 50% is represented by deciduous woods (Table 2).

**Table 2:** Area and frequency of occurrence (% of the experimental area) of the three vegetation units (VUs), rocky areas and feedwater.

Vegetation units	Surface (ha)	Frequency of occurrence (%)
Deciduous oak wood	30.3	55.8
Shrub-encroached grasslands	14.1	26.0
Grasslands	3.7	6.9
Rocky area	5.5	10.0
Feedwater	0.7	1.3

### Animals and GPS data

During the experimental period (January 2019–December 2020), a Sarda cow herd (N = 12, 442 ± 40 kg average live weight ± s.d., 11.6 ± 3.3 average years old ± s.d.) grazed the study area, with a stocking rate of 99 ± 2 kg live weight ha^−1^ (average ± s.d. between the 2 years of study). The Sarda breed is a small-to-medium-sized local breed, well-adapted to the harsh environment of Sardinian hilly and mountainous areas (28) Supplemantary data sheet 1. The stocking rate was held steady throughout the study duration in an attempt to avoid confounding stocking rate effects. During periods of shortage in pasture availability, the animals were supplemented with natural pasture hay [Dry Matter, DM 86.4%, Crude Protein 7.11% DM basis, Neutral Detergent Fiber 68.5% DM basis, Acid Detergent Fiber 4.5% DM basis, according to methods used by Cabiddu et al. (43)].

The experimental period, in which GPS-equipped collars were used to track animals, lasted for a total of 2 years and was divided into seven sampling periods (Table 1), two sampling periods for each season per year, except in 2020 when it was not possible to replicate autumn observation, due to Covid restrictions, which prevented people from leaving the house except for serious reasons. Each sampling period, four mature cows, randomly selected from the herd (consisting of 20 adult cows in total) and with multiple years of experience with the study area landscapes and herd management actions, were equipped with Knight GPS collars (43), programmed to collect and store GPS locations, date, Estimated Horizontal Position Error (EHPE, cm), and time every 3 min. By reason of the random selection of GPS-collared cows, their expressed variability in spatial behaviors was assumed representative of the herds. GPS collars were retrieved and data were downloaded, but resultant sample sizes, however, were unequal among experimental periods, due to some collar malfunction. Hence, for analysis, only cows with greater completeness of GPS data records were included in the data set. The Knight GPS collar uses the igotU GT-600^®^ GPS unit with a rechargeable battery. Details are provided in (44). As previously reported (45), the location error of igotU GPS logger is <10 m., although, as known, this value can vary depending on numerous factors. Data from GPS collars were then processed to remove gross GPS positioning error by using a GIS to exclude positions located outside the fence boundary of the study area. Moreover, according to the GPS collars manufacturer’s instructions, bad data were eliminated based on the animals’ speed and on drastic course changes. Tracking data recorded during the day of the collar placement and the day of removal were not used in our analyses. The fix rate (given by the ratio between the number of positions recorded and the scheduled number of positions) was calculated for each experimental period.

The ASSOC1 custom software (46) was used to determine if the collared cows had behaved spatiotemporally independent each other (i.e., were not associated). Associated behavior among collared cows would violate the independence assumptions of resource selection analyses conducted with these data (47, 48). In this study, the cows that spent >75% of their time separated by >25 m from each other were considered non-associated. Given the relative sizes of the study area and our RSF sampling units or plots (25-m radius; see below), this level of behavior independence was considered adequate for our objectives. The selected cows in this study were determined to be always non-associated, having spent at least 75% of their time separated from each other by more than 25 m during the study.

### Resource selection analyses

The effects of environmental factors on the cattle spatial distribution were evaluated using a negative-binomial (NB) regression approach (47), previously applied on other mountainous grazing lands (26, 27, 48). This regression model’s resource selection function (RSF) is defined by Manley et al. (49) but differed in some steps from logistic regression-based RSFs which are typically applied. Usually, logistic regression, which estimates the exponential RSF (49) relying on samples for used and available locations, considers a habitat unit as used, without assessing the intensity of use, regardless of whether that area was visited once or multiple times. Our NB-based approach assesses intensity of use (48) by developing an RSF model to estimate the probability of resource use as a function of environmental variables (26, 27, 48, 50, 51). Our modeling approach consisted of the following steps.

First step: a GIS [QGIS v. 3.10.14 “A Coruña,” (65)] was used to digitally create circular plots of 50 meters in diameter, randomly distributed throughout the fenced study area. Plots overlapping the fence boundary, with center points located <13 m of the fence, were removed, leaving 194 plots to be used in the analyses. The 50-m dia represented the best compromise between an area small enough to detect changes in animal movements but large enough to ensure multiple locations could occur in each unit. Too few plots tended to undersample the GPS positions present while too many plots failed to detect variability in animal use (51). After, the GIS was used to attribute, for each plot, the values of 12 predictor variables (Table 3). To this end, the following GIS thematic layers were created (48):

1. Vegetation type: A polygon layer was produced by analyzing an aerial photograph and identifying three vegetation units (*sensu stricto*, deciduous oak woods, shrub-encroached grasslands, and grasslands dominated by grasses), a rocky area, and a feedwater area (see above), which were then validated in the field using a GPS device.
2. Topography: An elevation layer at 10-m resolution was derived from a digital elevation model (DEM, 10 m),^3^ and raster layers for slope and other derived terrain index variables such as Topographic Ruggedness Index (TRI), Topographic Position Index (TPI), and aspect were obtained from the DEM with the Raster Analyst tool of QGIS. The topographic ruggedness index is a widely adopted measure of short-range roughness (52). Topographic position index (TPI) is a terrain classification where the altitude of each data point is evaluated against its neighborhood. If a point is higher than its surroundings, the index will be positive, as for example on hilltops, while the index will be negative for recessed features such as valleys.
3. Management: this layer contains locations of watering points and study area fence lines.

**Table 3:** Predictor variables used to develop the *a priori* set of candidate models for predicting resource selection patterns of mature beef cows on Mediterranean silvopastoral system.

Type	Predictor	Data type	Statistic or class	Units
Topographic	Elevation	Raster	Mean	Meters
	Slope	Raster	Mean	Degrees
	Aspect	Raster	North, East, South, West	NA
	Topographic Ruggedness Index (TRI)	Raster	Index	NA
	Topographic Position Index (TPI)	Raster	Index	NA
Vegetation	Cover type	Raster	Deciduous oak woods	sqm
	Cover type	Raster	Shrub-encroached grasslands	sqm
	Cover type	Raster	Grasslands	sqm
	Cover type	Raster	Feedwater	sqm
	Cover type	Raster	Rocky area	sqm
Distance	Fences	Vector	Minimum	Meters
	Watering/Supplements point	Vector	Minimum	Meters

From these vegetation, topographic, and management layers, predictive variable values in each plot were determined: cover percentages of deciduous oak woods, shrub-encroached grasslands and grasslands vegetation types, and feedwater and rocky areas in each plot were derived. Distance (m) to fences (26, distfences), and watering point (distwater) were determined by nearest-neighbor analysis of the distances between plot centroids and these linear and point features. Distance to point where the supplement was administered (distfeed) is practically equal to distance to water, the watering point and the “supplement point” being side by side. For this reason, from here on, this distance will be uniquely defined as distfeed. Quadratic terms for distance variables (i.e., distance to fences and water source) and for elevation and slope were also tested; models containing quadratic terms also contained the corresponding linear form of these variables (26).

Second step: GPS data for all collared animals of all sampling periods were pooled and subset by randomly selecting 75% of the locations for RSF model development and reserving the remaining 25% for model validation (48). In this way, the validation set (25% of locations) basically represents an external dataset to be used to validate the goodness of fit of the RSF model. Subsequently, counts were made of any GPS position within each plot. Counts of GPS positions located within these plots were tallied using a custom script written in the R programming language (53). A generalized linear model (negative-binomial NB regression, glm2 procedure of R software) was developed to estimate the probability of resource use as a function of environmental variables (RSF model) (26, 27, 48, 50, 51), to determine which combination of predictor variables was capable of providing an accurate prediction of resource-selection responses of cattle within the study area. Data from collared animals are pooled to estimate the population-level model. The relative number of cattle locations in the plots represents the dependent variable in this multiple regression analysis, modeling the probability of use as a function of environmental variables. This probability of cattle use was modeled as a continuous response variable in the NB model. The relative frequency of cattle use for each of the 194 plots was estimated, for both the model development (75% of data) and validation subsets, by counting the number of locations from each animal that occurred in the plot. A Pearson’s pairwise correlation analysis was conducted prior to NB regression development, to screen for multi-collinearity among predictor variables (|r| > 0.60). When collinearity was detected, only one variable of a collinear pair of variables in any one model was included. For any model which contained one of the variables from a collinear pair, another model was developed in which this variable was replaced with the other variable of the pair and both these models were retained for the final model selection process.

The following equations (1 and 2) (47) were used to estimate model coefficients:

$\ln\left(E\left[li\right]\right)=\ln\left(total\right)+\beta 0+\beta_{1}X_{1}+\ldots \ldots +\beta_{p}X_{p}$ (1).

which is equivalent to

$\begin{array}{l} \ln\left(E\left[li/total\right]\right)=\ln\left(E\left[\mathrm{Relative}\;{\mathrm{Frequency}}_{i}\right]\right)\\ \;\;\;\;\;\;\;\;\;\;\;\;\;\;\;\;\;=\beta 0+\beta_{1}X_{1}+\ldots \ldots +\beta_{p}X_{p} \end{array}$ (2).

where *li* is number of GPS locations within sampling unit i (i = 1, 2,…, 194); *total* is total number of GPS locations within the entire study area; β0 is an intercept term; β_1_, …, β*_p_* are coefficients for the predictor variables X1, …, Xp to be estimated; and E[.] denotes the expected value. The inclusion of an offset term, ln(*total*), serves to convert the integer counts of the response variable to relative frequency values, since to make inference to the relative frequency distribution of animal locations within the study area, also known as the utilization distribution (UD) (54), is often preferable to modeling counts (47). Since the offset term, ln(*total*), converts the integer counts of the response variable to relative frequency values, the NB regression estimates true probability of use (Resource Selection Probability Functions, RSPF) (49) for the sample of animals.

Third step: a set of over 500 candidate RSPF models, obtained following the procedure described before, composed of up to five predictor variables (Table 3), was identified *a priori* (55). Models were fitted to data from all collared animals of all sampling periods pooled together to develop a more general RSPF model (general RSPF model, objective 1), using the model development data sets (75% of total GPS positions acquired). Fitted models were then ranked on the basis of the Akaike’s information criterion (AIC) score (55, 56) and a short list of the 10 best-fitting models was developed. Prediction performance of the fitted models was evaluated with a Spearman correlation analysis (rs) using the GPS position data sets previously reserved for model validation (25% of total GPS positions acquired) (48, 51). The number of GPS locations within plots was counted in 20 equal-sized classes, from highest to lowest probability of cattle use. Afterwards, the analysis procedure assessed if the counts of GPS positions occurring within plots classified to each of these classes was related to the ranking of predicted probability of cattle use classes. When counts progressively increased with increasing class rank, we were in the presence of a strong correlation and successful model predictive performance (48). The Standard errors for the coefficients of this general cattle RSPF model and confidence intervals (90%) were calculated using a bootstrapping routine drawing 1,000 replicate samples from the model development data sets (48).

Fourth step: the 10 best-fitting negative-binomial regression models were fitted to data from collared animals across each different season to identify a model providing a robust prediction of cattle resource selection pattern for each different seasons (final model, objective 2) and thus having greater utility and impact. The models were fit using the model development data sets (75% of total GPS positions acquired each season). As before, prediction performance of the fitted models from each season was then evaluated based on a Spearman rank correlation analysis.

Subsequently, for each season, the estimated probability of use values, based on predicted resource-selection patterns derived from the final RSPF model, was assigned to each of the 194 plots representing the experimental area. Four classes (low, moderate, high, and very high probability of use) were identified. The classification was based on the quartiles of the distribution of predictions; consequently, each class contained approximately the same number of plots. In this way, the entire experimental area for each season was divided into four classes, depending on the lesser or greater probability of use. In order to characterize and ‘put a face’ to the four classes of predicted probability-of-use for each season, the Estimated Marginal Means of some predictive variables (distance to fences, distance to feed/water, elevation) were calculated through the use of a linear model with class as a fixed effect (emmeans procedure of R software) (53), to also highlight any significant differences in the values of these predictors among the four classes. Marginal effects plots, for each season, which display the estimated marginal effect of a variable holding the others constant, were realized to illustrate how predicted cattle use changed across the range of the observed data and across the seasons (objective 2).

Last step: to visualize in a map the cattle resource selection patterns for each season, a resultant raster layer at 25-m resolution, with a color gradient proportional to the predicted probability of cattle use, was produced in QGIS, with the darkest shade color representing the highest probability of use.

All statistical analyses were performed in the R Language (R software version 3.3.2) (53). Population-level RSPF model coefficients were reported as significant when bootstrapped 90% confidence intervals for coefficient estimates did not include zero. Although the experiment is replicated and controlled within the experimental area, it is still just a single, relatively small landscape, therefore the spatial scope of inference for this study is confined to the 54-ha study area.

## Results

Table 4 displays the number of experimental units (individual animals) represented in each experimental sampling period. Because of collar malfunctions and other contingencies and despite the fact four mature cows were selected for each period, the actual number of experimental units selected for analysis under this study, on the basis of completeness of GPS data record, was reduced. Nevertheless, the experimental units accounted for 25% of the herd, apart from the second winter sampling period, in which they accounted for 17%. Moreover, despite these occasional failures, the numerosity of the data analyzed for each sampling period is such that it did not impact the robustness of the data analysis (Table 4). After data selection, a total of 149,898 valid animal positions, recorded during the experimental periods, were kept (Table 4). The GPS fix rate for the different experimental periods is shown in Table 4. In this work, the low-cost GPS collar used showed an excellent GPS fix rate value for the first year of study. The Estimated Horizontal Position Errors (EHPE, cm), shown in Table 4, as calculated by igotU GT- 600^®^ GPS unit and referring to the experimental periods, were similar to what was reported in the literature.

**Table 4:** Fix rate value (average ± s. d.) of experimental periods, given by the ratio between the number of positions recorded and the scheduled number of fixes (480 positions every 24 h), Total valid animal positions, Estimated Horizontal Position Error (EHPE) calculated by igotU GT-600^®^ GPS unit and Experimental units (individual animals) represented in each experimental sampling period.

Seasons	Experimental periods	Fix rate (%)	Total valid animal positions, recorded during the experimental periods	EHPE (cm)	Experimental units
Winter	26/02/2019 to 03/03/2019	96.2 ± 3.3	27,594	897 ± 768	3
	14/12/2020 to 17/01/2021	51.1 ± 49.1	17,173	1,374 ± 734	2
Spring	06/04/2019 to 18/04/2019	97.9 ± 2.6	36,594	648 ± 504	3
	03/06/2020 to 08/07/2020	63.1 ± 39.7	20,385	1,515 ± 821	3
Summer	16/07/2019 to 24/07/2019	89.8 ± 2.0	11,639	1,553 ± 879	3
	05/08/2020 to 16/09/2020	69.5 ± 5.4	8,364	1,654 ± 979	3
Autumn	26/09/2019 to 16/10/2019	93.1 ± 2.2	28,149	1,446 ± 777	3

Table 5 lists the 10 best negative-binomial regression models fitted to all experimental periods, ranked on the basis of AIC score. Elevation, distance to fences (distfence), distance to feed (distfeed/water), and, as expected, feedwater area, seem to be, at least during the periods of the study, the most important factors affecting cattle resource-selection patterns for all experimental periods. From these 10 models, a model was selected as final cattle RSPF model (in bold in the table), based on its robustness and prediction performance when fitted to each season. This negative-binomial regression model contains five predictors (elevation, feedwater area, distance from feed, distance from fences, and TPI) but, for summer data, lacks the inclusion of the quadratic form of the distance to feed/water. This is because, when the identified final RSPF model (with quadratic term of distance to feed included) was applied to summer GPS data, it gave a lower rs value (0.65, data not shown). Although when discussing summer data we must use a different model (*sensu stricto*), we believe it should be emphasized that, even in this case, the importance of the same variables in predicting resource selection patterns of cows is confirmed. In our opinion, having identified these variables is still a significant result, especially for areas and for a livestock system for which, to our knowledge, such data are lacking. For convenience of exposition, we will henceforth refer to the two model variants, both with and without the quadratic term of the variable distance to feed, as the final cattle RSPF model. The prediction performances, in term of Spearman rank correlation scores (rs) of the final model when fitted to each season (each consisting of 2 years of data), are presented in Table 6.

**Table 5:** Top 10 cattle resource selection function (RSF) models, based on negative binomial regression, fitted to all experimental data, selected from an *a priori* set of over 500 candidate models based on Akaike’s information criterion (AIC) fit scores, and value of Spearman rank correlation scores.

Ratings	Models	AIC	Spearman rank correlation scores (rs)
1	**y = feedwater + elevation + elevation**^**2**^ **+ distfeed + distfeed**^**2**^ **+ distfence + distfence**^**2**^ **+ TPI**	18266.1	0.71
2	y = feedwater + elevation + elevation^2^ + distfeed + distfeed^2^ + distfence + distfence^2^ + grasslands	18330.4	0.77
3	y = wood + feedwater + elevation + elevation^2^ + distfence + distfence^2^ + distfeed + distfeed^2^	18338.0	0.68
4	y = distfeed + distfeed^2^ + distfence + distfence^2^ + slope + slope^2^ + TPI + elevation + elevation^2^	18338.1	0.58
5	y = wood + TPI + elevation + elevation^2^ + distfence + distfence^2^ + distfeed + distfeed^2^	18339.4	0.69
6	y = feedwater + elevation + elevation^2^ + distfeed + distfeed^2^ + distfence + distfence^2^ + rocce	18342.9	0.69
7	y = feedwater + distfence + distfence^2^ + elevation + elevation^2^ + distfeed + distfeed^2^ + aspect	18345.7	0.75
8	y = feedwater + elevation + elevation^2^ + distfence + distfence^2^ + slope + slope^2^ + distfeed +distfeed^2^	18347.4	0.64
9	y = feedwater + TRI + elevation + elevation^2^ + distfence + distfence^2^ + distfeed + distfeed^2^	18349.1	0.59
10	y = feedwater + TRI + elevation + elevation^2^ + distfence + distfence^2^ + distfeed + distfeed^2^	18350.7	0.74

^1^Feedwater is the area characterized by the presence of water point and the feed supplement (prop); elevation is terrain elevation (m); distfeed is distance to water point or supplement point (m); distfence is distance to fences (m); TPI is Topographic Position Index (index); grasslands are the areas classified as grassland; wood are the areas classified as deciduous oak woods; Slope indicates terrain slope (deg); TRI is Topographic Ruggedness Index (index); aspect is terrain aspect (cardinal direction) and superscript “2” indicates values have been squared. Model in bold font was selected as the final cattle (RSPF) model.

**Table 6:** Spearman rank correlation scores (rs) quantifying the prediction success of the final cattle resource selection probability function (RSPF) model when fitted across seasons.

Experimental periods	Spearman rank correlation score (rs)
Winter	0.77
Spring	0.88
Summer	0.80
Autumn	0.90

The relative importance of the individual predictors within the models varied among these different cases, but the models were still fairly effective at predicting the relative probability of cattle use within this broader, more diverse scope. Spearman rank correlation analysis of the final cattle RSPF model, using the GPS data sets previously reserved for model validation, yielded fairly good prediction success (Table 6), showing a sufficiently robust predictive performance across a discrete space–time range.

The range of values of predictors in the final RSPF model, representing the conditions within which the model could make a good estimate of relative probability of cattle use, is shown in Table 7.

**Table 7:** Range of values of predictors used in the finel RSPF model for predicting the relative probability of cattle use in a mediterranean silvopastoral area.

Predictors	Means±s.d.	Range of values
Elevation (m)	695 ± 15	660–731
Feedwater (% coverage)	1.4 ± 9.5	0–96.2
Distance to feed/water (distfeed, m)	600 ± 335	15–1,275
Distance to fences (distfence, m)	104 ± 57	16–225
TPI (NA)	−0.004 ± 0.081	−0.213–0.413

**Figure 2:**
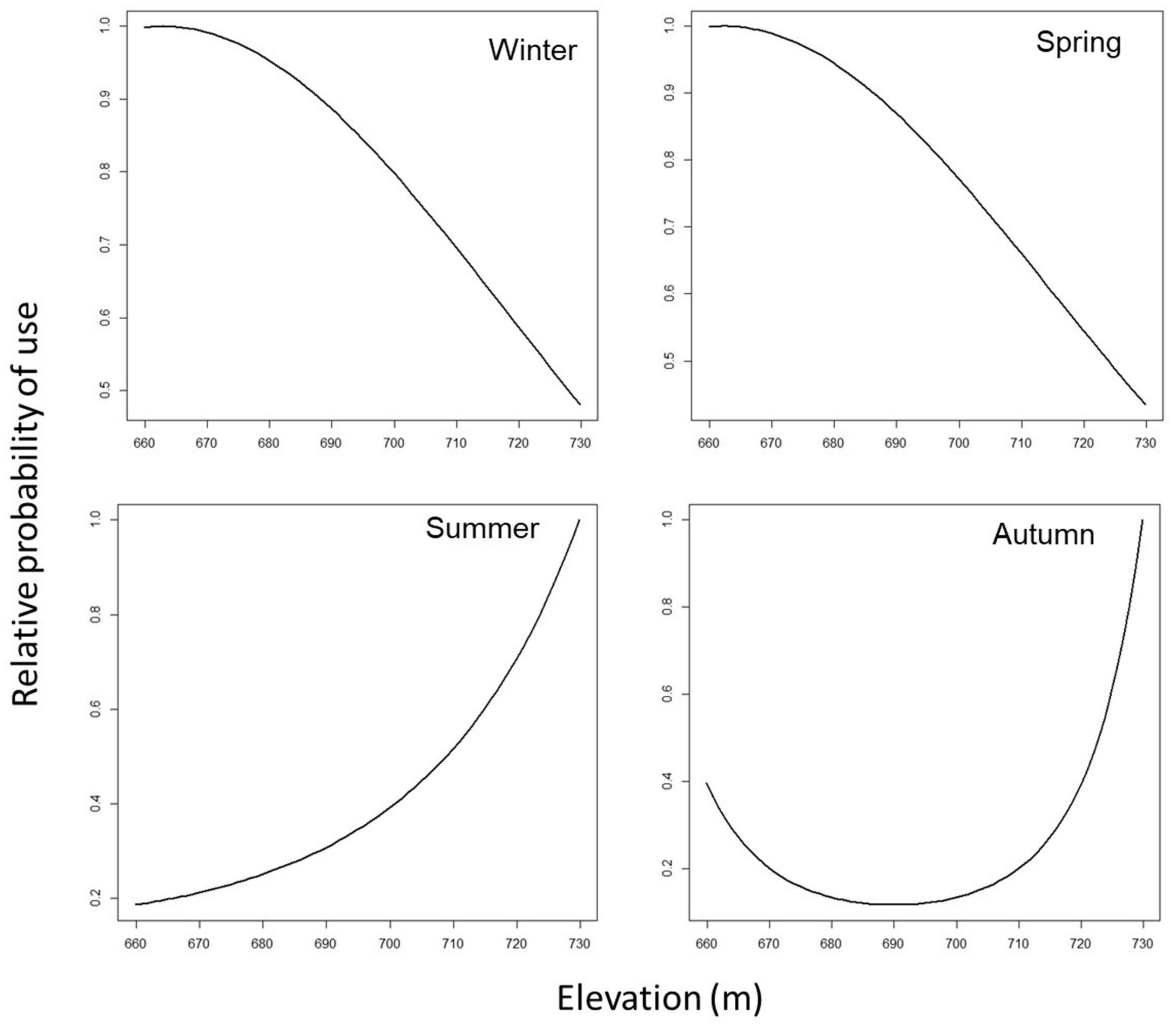
Predicted cattle relative probability of use response to the distance to fences (m) predictor, in the final cattle resource selection function model, for each season in Mediterranean mountainous silvopastoral area.

**Figure 3:**
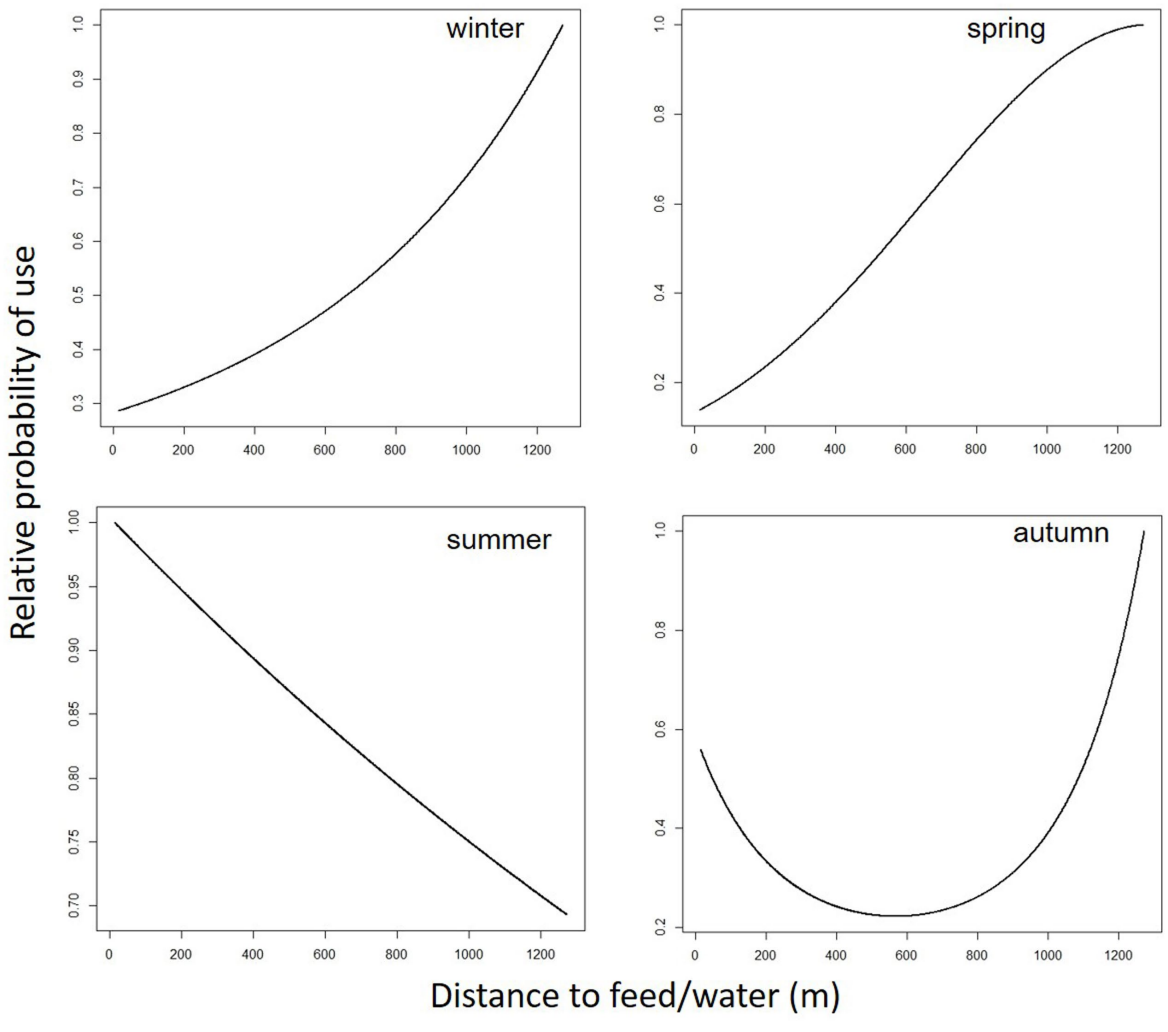
Predicted cattle relative probability of use response to the distance to feed/water (m) predictor, in the final cattle resource selection function model, for each season in Mediterranean mountainous silvopastoral area.

**Figure 4:**
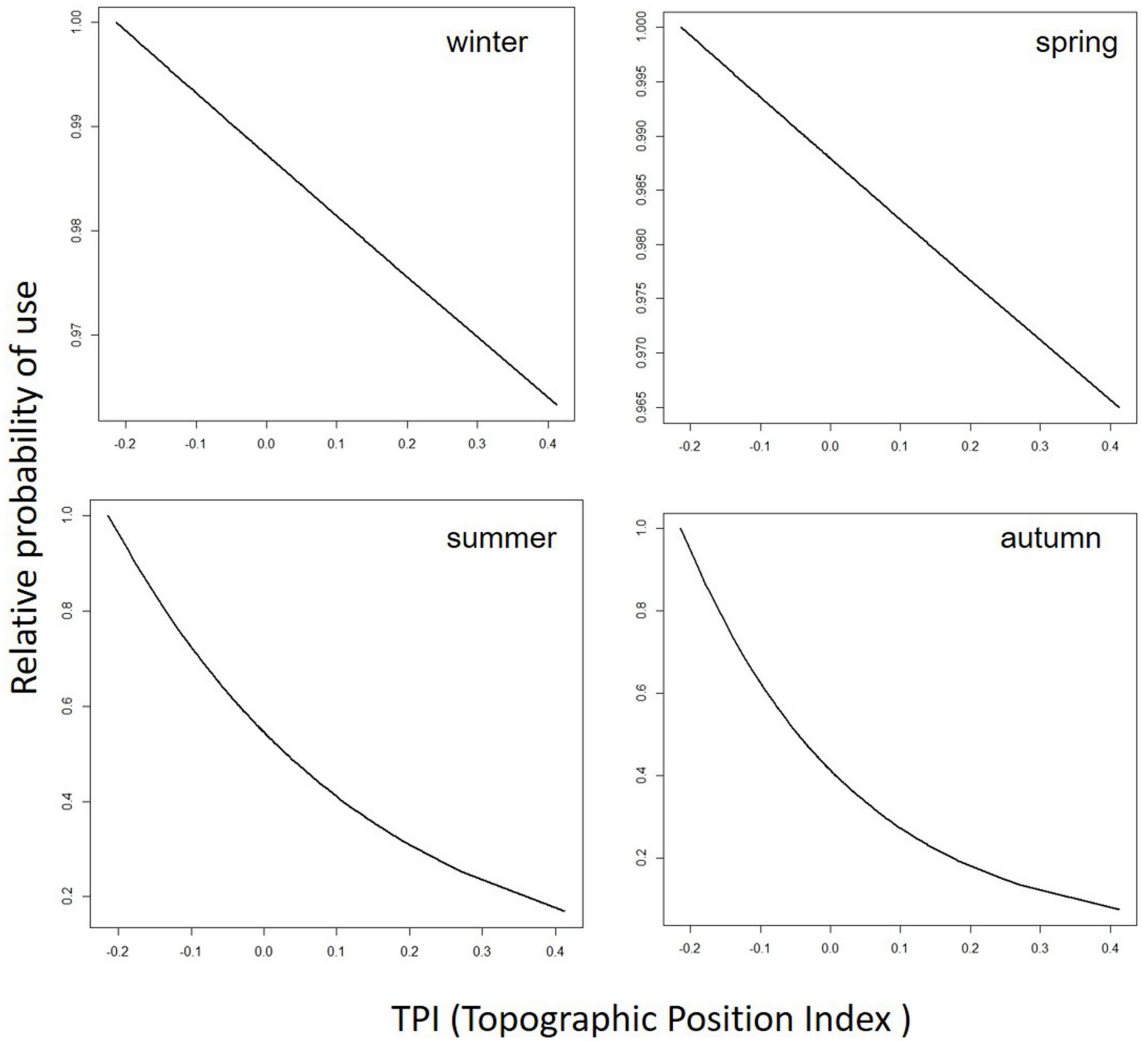
Predicted cattle relative probability of use response to the topographic position index (TPI) predictor, in the final cattle resource selection function model, for each season in Mediterranean mountainous silvopastoral area.

**Figure 5:**
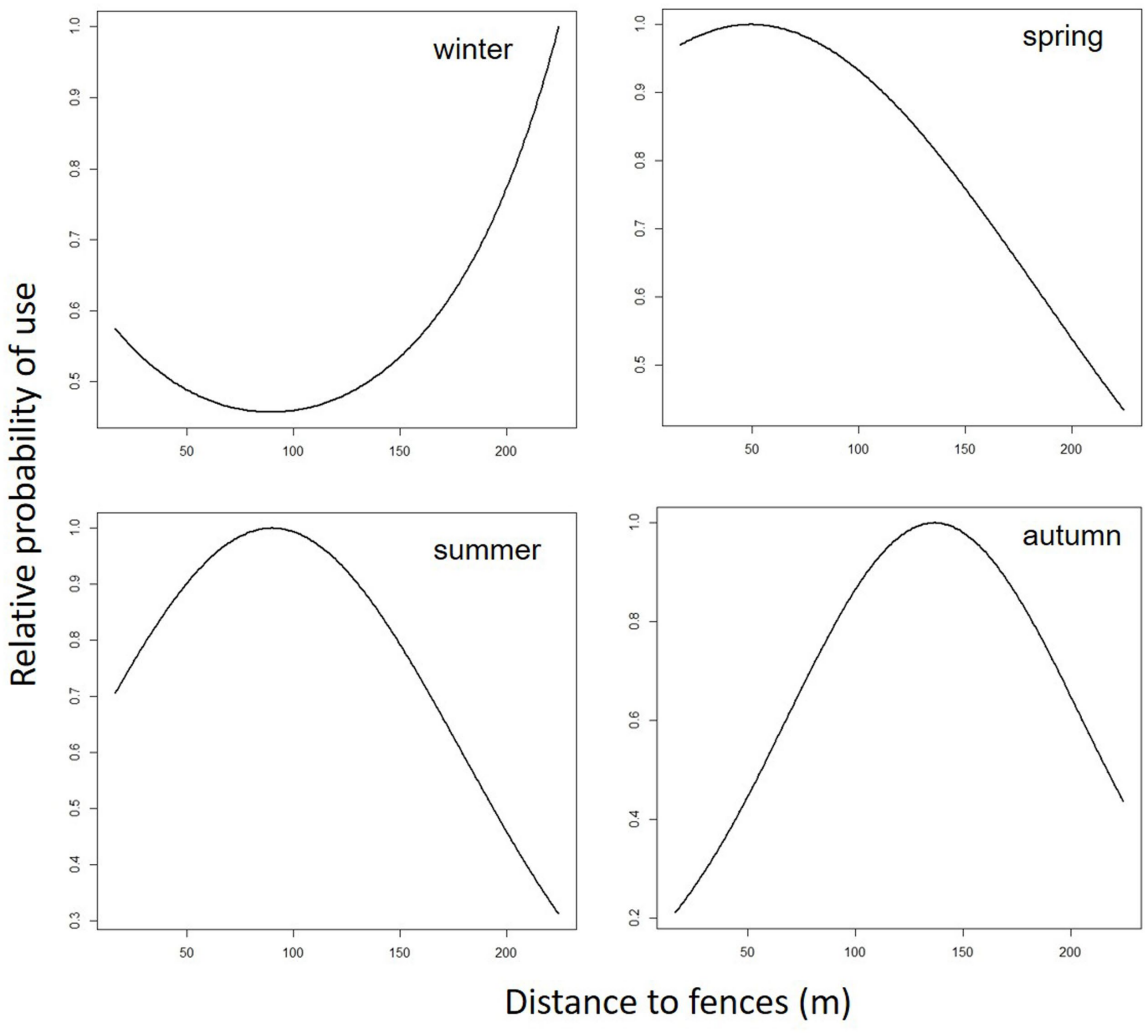
Predicted cattle relative probability of use response to the elevation (m) predictor, in the final cattle resource selection function model, for each season in Mediterranean mountainous silvopastoral area.

Table 8 lists the coefficient estimates and the 90% confidence level (CL) of the final cattle resource selection function (RSPF) model fit across seasons. As a general rule, in the regression model used in this work (negative binomial regression), the meaning of the coefficients of the variables is that for each one-unit increase of a variable (e.g., TPI in winter-RSPF model, with coefficient value −5.99E-02), the log count of dependent variable increases (or decrease if the coefficient is negative) by −5.99E-02. The coefficients are easily interpreted through odds ratios and marginal plots (Figures 2–5). The odds ratio for TPI indicated that probability of cattle use was expected to change by [exp(−5.99E-02) -1] X 100% = − 5.81% for every increase of one unit in TPI index (47).

**Table 8:** Fitted coefficients and statistics of the final cattle resource selection function (RSPF) model predicting the relative probability of cattle use in a Mediterranean silvopastoral area applied to each season.

Predictor	Estimate	Lower 90% CL	Upper 90% CL	*p* value
Winter
(Intercept)	−7.76E+01	−3.27E+02	1.42E+02	0.52
feedwater	3.09E-03	2.53E-03	3.99E-03	**< 0.001**
elevation	2.16E-01	−4.14E-01	9.36E-01	0.53
elevation2	−1.63E-04	−6.79E-04	2.88E-04	0.51
distfeed	7.19E-04	−3.69E-04	1.98E-03	0.36
distfeed2	2.13E-07	−7.15E-07	1.01E-06	0.71
distfence	−7.68E-03	−1.34E-02	−1.68E-03	0.07
distfence2	4.30E-05	1.72E-05	6.81E-05	**0.02**
TPI	−5.99E-02	−1.32E+00	1.53E+00	0.94
Predictor					Estimate	Lower 90% CL	Upper 90% CL	*P* value
Spring
(Intercept)	−8.68E+01	−2.50E+02	6.19E+01	0.37
feedwater	1.84E-03	1.52E-03	2.15E-03	**< 0.001**
elevation	2.42E-01	−1.88E-01	7.17E-01	0.39
elevation2	−1.82E-04	−5.22E-04	1.27E-04	0.36
distfeed	3.12E-03	2.08E-03	4.10E-03	**< 0.001**
distfeed2	−1.20E-06	−1.90E-06	−4.36E-07	**0.01**
distfence	2.70E-03	−2.32E-03	8.54E-03	0.44
distfence2	−2.73E-05	−5.25E-05	−4.67E-06	0.07
TPI	−5.70E-02	−1.06E+00	1.09E+00	0.92
Predictor					Estimate	Lower 90% CL	Upper 90% CL	*P* value
Summer
(Intercept)	6.52E+01	−2.54E+02	4.10E+02	0.67
feedwater	2.19E-03	1.51E-03	2.97E-03	**< 0.001**
elevation	−2.30E-01	−1.22E+00	6.84E-01	0.60
elevation2	1.82E-04	−4.71E-04	8.82E-04	0.56
distfeed	−2.92E-04	−7.15E-04	2.09E-04	0.30
Distfeed2				
distfence	1.16E-02	2.78E-03	2.14E-02	**0.04**
distfence2	−6.45E-05	−1.04E-04	−2.78E-05	**0.01**
TPI	−2.84E+00	−4.52E+00	−1.22E+00	**0.003**
Predictor					Estimate	Lower 90% CL	Upper 90% CL	*P* value
Autumn				
(Intercept)	6.36E+02	3.47E+02	8.88E+02	**< 0.001**
feedwater	−6.93E-04	−1.52E-03	2.66E-05	**0.05**
elevation	−1.86E+00	−2.58E+00	−1.04E+00	**< 0.001**
elevation2	1.35E-03	7.59E-04	1.86E-03	**< 0.001**
distfeed	−3.43E-03	−4.83E-03	−1.75E-03	**< 0.001**
distfeed2	3.02E-06	1.89E-06	3.97E-06	**< 0.001**
distfence	2.94E-02	2.27E-02	3.63E-02	**< 0.001**
distfence2	−1.08E-04	−1.36E-04	−7.81E-05	**< 0.001**
TPI	−4.14E+00	−5.48E+00	−2.90E+00	**< 0.001**

While the coefficient estimates are based on pooled GPS data, the 90% confidence level (CL) were calculated from bootstrapped samples from individual cattle data sets. p values significant at the 0.05 alpha level are highlighted in bold font.

From the predicted resource-selection patterns of cattle, originated from the application of the final RSPF model to the GPS data of each season, an estimated probability of use values was assigned to each of the 194 plots representing the experimental area. On the basis of this estimated probability of use, the plots were then divided into four classes, representing the low (class 1), moderate (class 2), high (class 3), and very high probability of use (class 4). Table 9 showed the estimated marginal means of elevation, distance to fences, and distance to feed/water characterizing the four classes in the different seasons.

**Table 9:** Estimated marginal means (aka Least-Squares Means) values (emmeans± standard error, SE) of predictor variables (distance to water/feed, distance to fences and elevation) of the predicted probability-of-use classes, estimated from the final RSPF model applied to the different seasons.

		Distance to water/feed (m)	Distance to fences (m)	Elevation (m)
		emmean±SE	emmean±SE	emmean±SE
Winter	1 class	375.68 ± 13.56^a^	82.62 ± 2.77^a^	700.92 ± 0.71^d^
	2 class	422.51 ± 13.42^a^	99.35 ± 2.75^b^	689.50 ± 0.71^a^
	3 class	751.92 ± 13.56^b^	109.05 ± 2.77^b^	695.87 ± 0.71^c^
	4 class	846.81 ± 13.42^c^	124.08 ± 2.75^c^	693.11 ± 0.71^b^
Spring	1 class	245.52 ± 11.99^a^	130.77 ± 3.10^c^	688.70 ± 0.84^a^
	2 class	480.89 ± 11.87^b^	101.61 ± 3.07^b^	697.22 ± 0.83^b^
	3 class	703.56 ± 11.99^c^	109.34 ± 3.10^b^	697.48 ± 0.84^b^
	4 class	963.30 ± 11.87^d^	74.36 ± 3.07^a^	695.78 ± 0.84^b^
Summer	1 class	479.58 ± 21.11^a^	136.97 ± 3.38^c^	687.71 ± 0.80^a^
	2 class	602.54 ± 20.89^b^	102.94 ± 3.35^b^	688.54 ± 0.80^a^
	3 class	679.53 ± 21.11^c^	96.41 ± 3.38^b^	696.19 ± 0.80^b^
	4 class	635.91 ± 20.89^bc^	79.62 ± 3.35^a^	706.70 ± 0.80^c^
Autumn	1 class	489.71 ± 26.52^a^	88.58 ± 4.60^a^	691.67 ± 1.17^a^
	2 class	512.89 ± 26.25^a^	119.96 ± 4.55^c^	690.00 ± 1.16^a^
	3 class	640.18 ± 26.52^b^	110.39 ± 4.60^bc^	696.78 ± 1.17^b^
	4 class	754.18 ± 26.25^c^	96.31 ± 4.55^ab^	700.78 ± 1.16^b^

Different lowercase letters indicate significant differences within the same season (*p* < 0.05).

### Specific responses to predictors

Predicted cattle responses to the individual predictors within the final model were then evaluated across the four seasons. Figures 2–5 show the marginal effect plots of the predicted relative probability of use response to elevation (Figure 2), distance to fences (Figure 3), distance to feed (Figure 4), and TPI (Figure 5). As stated before, marginal effects plots for each season display the estimated marginal effect of a variable holding the others constant. Predicted cattle use decreased in a parabolic trend with increasing elevation (Figure 2) in winter and spring, whereas the opposite occurred in summer. In autumn, the probability of use initially declined with elevation, up to 690 meters, and then increased with increasing elevation. Cattle use was predicted to exhibit a parabolic fashion in relation to distance to fences but with an inverse trend in winter compared to spring, summer, and autumn (Figure 3). As shown in Table 9, increase occurred at distances above 100 meters from fences in winter. In spring, use declined at distances from fences greater than 50 meters; in summer and autumn, the use peaked at approximately 90 and 130 meters from fences, respectively. A gentle curvilinear increase in predicted use with distance to water occurred in winter and spring, although, in the latter, there is a glimpse of a decline in use beyond 1,200 m. from water. While in summer the effect of distance to water on cattle use was linear, with a decline in the probability of use as distance from the water increases, in autumn the trend was parabolic. An initial decline in use as the distance from water increases, reaching a minimum at a distance of about 600 meters, is followed by an increase in use, with a maximum beyond 700 m (Table 9). In winter and spring TPI did not appear to play an important role in the probability of use by animals, while in summer and autumn an increase in TPI value leads to a decrease in the probability of use.

### Visualization of cattle resource selection patterns

**Figure 6:**
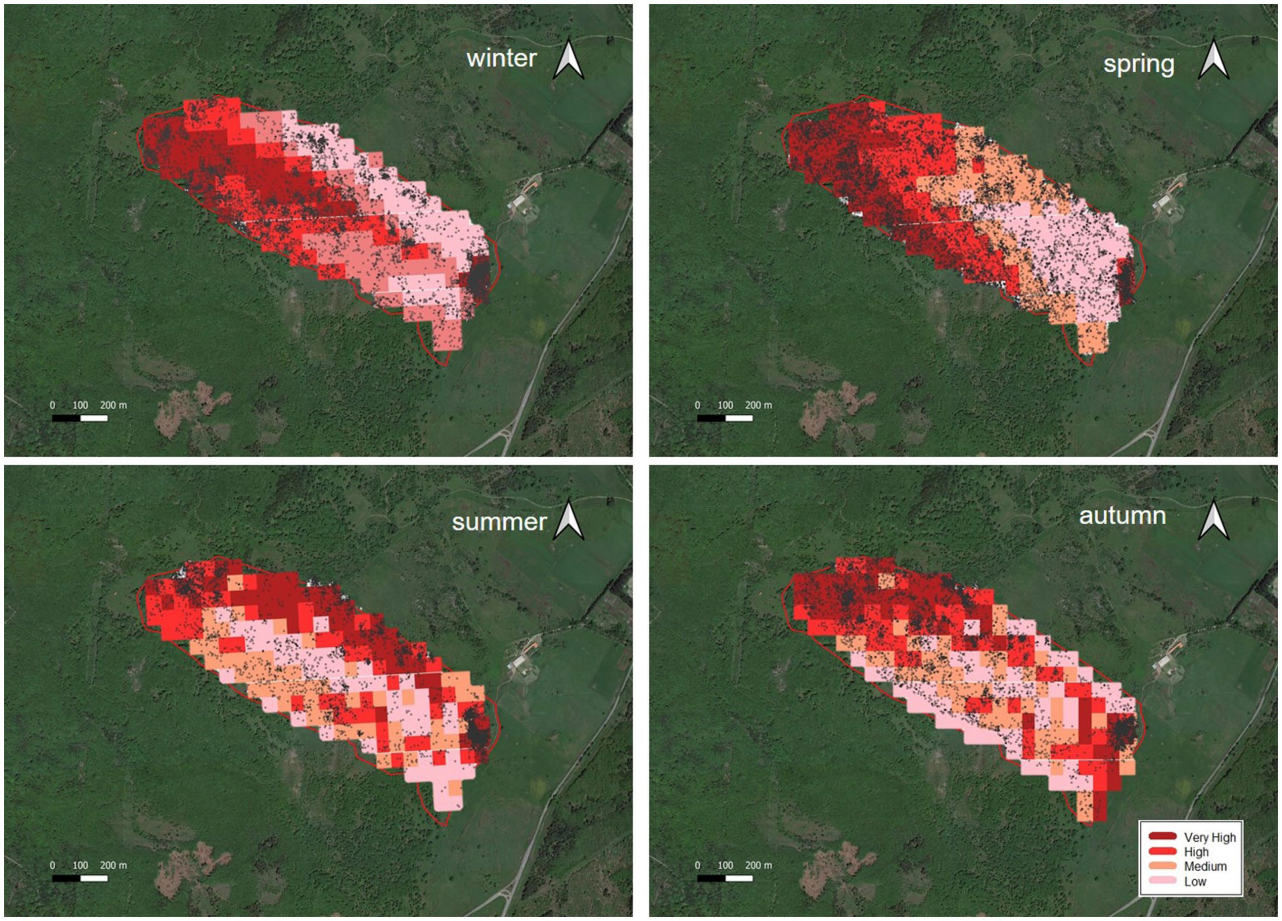
Seasonal prediction use classification map of the cattle resource selection pattern of Sarda cattle in the mediterranean silvopastoral experimental area, as derived from the application of the final RSPF model to the GPS data of different season. The darkest shade color represents the zones with the highest probability of cattle use*. The dark dots represent the cows’ positions. *Probability of cattle use: Very high: from 1.7 to 4.3% depending on the season. High: from 1 to 2.4%. Medium: from 0.7 to 1.1%. Low: from 0.4 to 1%.

To visualize changes in habitat use, it is helpful to map model predictions and identify key habitats within the same study area at different points in time (47). Figure 6 shows the resource selection pattern of Sarda cattle in different seasons, as derived from the application of the final RSPF model to GPS data. In the map, the darkest shade color represents the zones with the highest probability of cattle use. The dark dots represent the cows’ GPS localization.

## Discussion

Cattle require adequate amounts of usable resources to sustain themselves. The need to identify resources used by animals, quantify the availability of those resources, and determine which resources are selected more often than others is of the utmost important in efforts to provide information about the nature of animals, how they meet their requirements for survival, and how best to manage livestock (49). To compare the amount of used habitat with the amount of available habitat, resource selection functions models are employed. When a habitat is used by animals more than expected, with respect to its proportion across the landscape, the habitat is assumed to be selected (57). Comparing the characteristics of available locations with those used through an exponential RSF (49) constitutes the basis of the resource selection or use (47). RSFs are employed to estimate and predict spatial distributions and resource use by animals (58, 59). To develop these models, data from a set of used points and a set of available points are needed to identify environmental variables that best predict resource selection by animals (49, 60). GPS is a good tool to collect animal location at fine spatiotemporal scales. In this work, the low-cost GPS collar used showed excellent GPS fix rate values for the first year of study (Table 5). The build-up of dust and various debris on the GPS loggers due to the home-made, not hermetically sealed boxes containing the GPS, could have led to a general reduced efficiency of the devices, explaining the unfavorable results of second year. Some physical damage to the electronics that occurred over time could also be suspected, causing intermittent electrical shorts. iGotU units are not especially watertight and, thus, the electronic eventually become exposed to moisture and corrosion.

Although caution should be exercised in generalizing to other silvopastoral areas, the results obtained in this work and the satisfactory Spearman correlation scores from the final RSPF model applied to different seasons (Table 6) indicates resource selection function to be a powerful predictive model. As expected, the watering and feeding point and the distance to this point were important factors affecting cattle resource selection patterns in a Mediterranean silvopastoral area. Somewhat less obvious is the importance, as predictors, of elevation and distance to fences. The TPI factor was found to be an important predictor, especially in summer and autumn. The relative importance of each predictor within the model varied depending on the seasons, as shown by the sign of the coefficient of some predictors (Table 8), demonstrating the RSPF model’s ability to interpret changes in animal behavior at different times of the year. For summer data, the inclusion, among the model predictors, of the linear form of the distance from the water, increased the goodness of fit of the model (as measured by Spearman’s coefficient), compared with the general RSPF model (used for all other seasons) that included the quadratic form of the distance from the water. While admitting that, strictly speaking, this is a different model from the general RSPF model, it must be acknowledged that the variables that constitute the model are the same as those in the general RSPF model and we believe that identifying these as the major determinants of animal movement is an important achievement. Although the identified RSPF model provides a robust prediction across different seasons (the main purpose of the work) by jointly using different predictors, and conscious of representing the mechanism under study in a simplified manner, below we will analyze the response of cattle to individual predictors, with the purpose of identifying the causes of the predicted response of animals to predictors.

### Elevation

The predicted cattle relative probability of use response to the elevation showed an opposite trend in winter and spring compared to summer and autumn (Figure 2). In the first case, higher quotas correspond to a lower probability of utilization. In winter this behavior could be due to the fact that higher areas are more exposed to winds and therefore tend to be colder. Animals tend to frequent the central part of the paddock, further away from the fences (see Figure 3, distance to fences and Table 9), as it is more sheltered and characterized by intermediate elevations (690–696 m a.s.l.), as also told by the average elevation value that characterizes the class with the highest probability of use (Table 9). In spring, on the other hand, the temperature aspect is less important, and the differences in elevation between the higher- and lower-probability of use classes are smaller (Table 9). Figure 6 shows that cattle, in spring, tend to frequent an area on the west side of the pasture, not far from fences (Table 9, distance to fences, 4 class, spring), characterized by medium-high altitudes (692–699 m). The area during the study period experienced heavy thinning of trees, damaged by the passage of a tornado, leaving some clearings that in the spring may have constituted grazing areas sought by livestock. In summer and autumn, higher quotas correspond to a higher probability of use. In these two seasons, as opposed to winter, the search for cooler areas may have resulted in this behavior. According to other authors (61, 62), this shade-seeking and its relationship to temperature are well-known driving factors in areas utilized by livestock.

### Distance to fences

In winter, cattle use was predicted to exhibit a parabolic trend in relation to distance to fences, with a minimum value on 100 m from fences (Table 9) and then increasing at greater distances. This confirms the tendency of cattle to use in winter the central area of the pasture, at greater distances from the fences. In spring, summer, and autumn, the cattle showed an inverse trend (Figure 3); in spring the predicted probability of use is higher relatively close to fences (approximately around 50 m; Table 9; Figure 3), probably related to the clearings created after the tornado, as stated before. In summer and autumn, on the other hand, the area with the highest probability of use was at an intermediate distance from the fences (approximately 85 and 130 m., respectively, Figure 3). This area, located in the eastern part of the pasture, about 80 m. from the fences (Figure 2; Table 9), is the highest point of the pasture (722 m a.s.l.) and, as mentioned before, probably represents the coolest area in summer. Moreover, in this season, the animals avoided the central area of the pasture, furthest from the fences, due to the high presence of bracken in this area. This confirms what was stated by Acciaro et al. (28), who found that the shrub-encroached grassland areas were avoided in the summer, in conjunction with the maximum presence of bracken (*Pteridium aquilinum* (L.) Kuhn), which are unpalatable species containing antinutritional factors (63). In autumn, the area with the highest probability of use was predicted to be in the northwest of the pasture, at higher elevation, likely characterized by slightly higher pasture quality compared to warmer areas where, in a Mediterranean environment, the grass is almost completely dry at that time. In the autumn, higher areas may have retained better foraging quality. This area is precisely located about 110–140 meters from the fences (Figure 6).

### Distance to water/feed

As mentioned before, the distance to water and to supplemental feed are practically the same. While a gentle curvilinear increase in predicted use with distance to this point occurred in winter, in spring, this trend is less pronounced, showing a peak at about 1,200 meters and then beginning a decrease in the probability of use. The two trends would seem to highlight how cows, in winter, after drinking (and eating in the case of hay administration, which is usual for this season) move in search of more distant areas. In spring, on the other hand, the distances to the watering point do not go beyond 1,200 meters, an aspect that is in line with Cowley et al. (64), who hypothesize that at these distances from the water point, pasture use efficiency drops to 40%. In summer, the RSPF model applied includes the exclusion of the quadratic form of the distance-to-water variable, and consequently the plot trend was linear. More precisely, the probability of use increases as the distance from the water point decreases. This is expected in the hottest season. The linear trend of the graph (and thus the exclusion of the quadratic form of the distance-to-water predictor from the model) seems to emphasize the utmost importance of water in this season. In autumn the trend was parabolic. An initial decline in use as the distance from water increases, reaching a minimum at a distance of about 600 meters, is followed by an increase in use, with a maximum above 900–1,000 meters (Table 9; Figure 4). One possible explanation is related to the fact that, in autumn, the only areas where cows can find herbage at a less advanced stage (and therefore of better quality) are at the higher elevation zone, which is at a greater distance from the watering point. This would explain the parabolic pattern of the predicted cattle relative probability of use response to the distance to feed/water in the autumn, with greater probability of presence both near the watering point and at distances of more than 800–900 meters.

### TPI

In winter and spring, the TPI variable appears to have little influence on the prediction of the probability of use, as evidenced by the small differences in the relative probability of use as the TPI value changes. More complicated is to explain the response in predicted use to TPI in summer and autumn, when an increase in the probability of use corresponds to a decrease in the value of TPI, such as that cows prefer to frequent topographically lower areas. The low elevation of the water point (665 m) could be one of the causes of this result, at least for the summer period. Whereas, as with other predictors, the predictive role of TPI alone is limited, when together with the other factors within the RSPF it allows a prediction with good accuracy of the spatial distribution of cattle.

Overall, the RSPF model was able to predict, with satisfactory ability, the resource selection of Sarda cattle across a discrete space–time range, represented by the different seasons. The study suggests that basic habitat variables, such as elevation, distance to fences, and distance to water can successfully predict seasonal habitat use of Sarda cattle in this open environment. Understanding the role of these variables on cow’s distribution could give land managers relevant information for management of livestock to address both conservation and production objectives from these ecosystems. Development of models predicting livestock grazing distribution could be used to guide decisions on a wide range of management actions, altering habitat attributes to change livestock distribution, including modification of fencing, putting in place water sources or rewards such as molasses supplement, and installing artificial shade. Moreover, breeds of livestock differ in use of foraging areas of varying slope or distances from water. A tool that can predict livestock spatial behavior can also be used in the identification of breed with the desired habitat-use characteristics, improving livestock distribution in pastures.

## Conclusion

Results of this investigation try to improve our understanding of habitat selection by cattle within heterogeneous Mediterranean silvopastoral area. The GPS data allowed developing a model (Negative Binomial regression model) able to predict the probability of resource use of Sarda cattle grazing a silvopastoral area as a function of environmental variables (Resource Selection Probability Function model). In the foreseeable future, modeling efforts could also be extended by including other abiotic (i.e., soil type) or biotic variables such as herbage quantity and quality, perhaps through remotely obtained spectral indices of vegetation.

## Data availability statement

The raw data supporting the conclusions of this article will be made available by the authors, without undue reservation.

## Ethics statement

The animal study was approved by Ethical Committee on Animal Experimentation (OPBA, No. 2190/2019). The study was conducted in accordance with the local legislation and institutional requirements.

## Author contributions

MA: Conceptualization, Data curation, Formal analysis, Investigation, Methodology, Software, Supervision, Visualization, Writing – original draft, Writing – review & editing. MP: Formal analysis, Methodology, Software, Supervision, Validation, Visualization, Writing – review & editing. MD: Conceptualization, Data curation, Methodology, Supervision, Validation, Visualization, Writing – review & editing. MS: Funding acquisition, Project administration, Supervision, Validation, Writing – review & editing. VG: Methodology, Supervision, Validation, Writing – review & editing. GL: Formal analysis, Funding acquisition, Methodology, Project administration, Resources, Writing – review & editing. PC: Conceptualization, Data curation, Formal analysis, Methodology, Software, Supervision, Validation, Visualization, Writing – original draft, Writing – review & editing.

## Footnotes

^1^https://op.europa.eu/en/publication-detail/-/publication/22542b64-c501-11e7-9b01-01aa75ed71a1/language-en/format-PDF/source-search

^2^http://www.sar.sardegna.it/

^3^http://www.sardegnageoportale.it/webgis2/sardegnamappe/?map=download_raster
