# Supplementary material for: Resource selection by Sarda cattle in a Mediterranean silvopastoral system
Source: Front Vet Sci. 2024 Mar 7;11:1348736. doi: 10.3389/fvets.2024.1348736 (PMC10954795; doi:10.3389/fvets.2024.1348736)
Supplement: Supplementary file 1 [file Data_Sheet_1.pdf]

The Sarda breed

The presence of bovine species in Sardinia is documented since the Neolithic age (2000-1800 b.C.) by the introduction in the Island of animals type macrocero, with horns directed backwards, attributable to ancestor *Bos macrocerus*, that probably was of North-Africa, Syrian or Iberian origin. These animals would have been improved through the introduction (900-400 b.C) of brachicero cattle with horns directed laterally, that was of Punic origin.

Until the middle of the IX century this bovine species (Sarda breed) was characterized by highly variable morphological, reproductive and productive features, at least from a phenotypic point of view; this variability has been reported as a consequence of the great variability of the environment where they are reared (i.e. altimetry, orography…). Nevertheless, some mention can be given of its morphological characteristics.

The Sarda breed is small-sized, variously pigmented, with almost straight profile, eyes large and lively, ears of medium size, snout broad, black to pinkish in color, jaws strong and sturdy, horns of various shape, size and direction. This breed has long, light neck, withers more prominent in males, light shoulders, medium-sized chest, strong, dry limbs, regular loins and strong feet with hard nails. The Sarda breed has an almost straight dorsal line with a raised sacral spine and strong loins. The rump is slightly sloping. The tail is long with high attachment and abundant bow. The loins are regular with thigh dry in females and muscular in males. The feet are correct with hard nails. Regarding the sexual characteristics: udder of varying shape and size, well attached with quarters regular and symmetrical. The teats are medium in size and well arranged, perimammary veins usually not very evident and subcutaneous-abdominal veins evident.

The Sarda breed, characterized by hardness and resistance and well adapted to the different environments of the Island, was raised mainly for milk production and demonstrates a poor attitude either to work in heavy soil plains (because of its small size and under-developed chest), or to meat production (because of the poor growth rate and the low dressing percentage).

With changing economic conditions and the enormous specialization of modern dairy breeds, today the residual aptitude of the Sarda breed is meat production. Nowadays, this native breed is exploited for the calf production, obtained by crossbreeding with bulls of the large-frame beef breed, mainly Charolais and Limousine, in order to improve meat production and carcass quality of calves.

This native and small breed, thanks to its excellent rustic and frugal qualities, has maintained its original characteristics in the most unfavorable areas. To date, it is present on the island with fragmented populations that take different names (Istringata, Pettiatza, Bertigazza, Sorgolina, Planaria or Montagnola) based on coat color and stature, in the less favoured hill and mountain regions of Island (Limbara, Gennargentu, Sette Fratelli-Serpeddì, Linas) where traditionally is used this small-frame cows that grazed upland pastures and forests all year around in suckler-cows system

These data and some pictures illustrating the Sarda breed can be found on the Region of Sardinia website: https://www.biodiversitasardegna.it/laore/it/agrobiodiversita/repertorio-regionale/risorsa/Vacca-sarda/
